# Supplementary figures and images for: Hospital Antibiotic Consumption before and during the COVID-19 Pandemic in Hungary
Source: Antibiotics (Basel). 2024 Jan 20;13(1):102. doi: 10.3390/antibiotics13010102 (PMC10812576; doi:10.3390/antibiotics13010102)

Figure S1: Trend analysis of antibiotic utilisation expressed as DDD per 100 patient-days (DHPD)

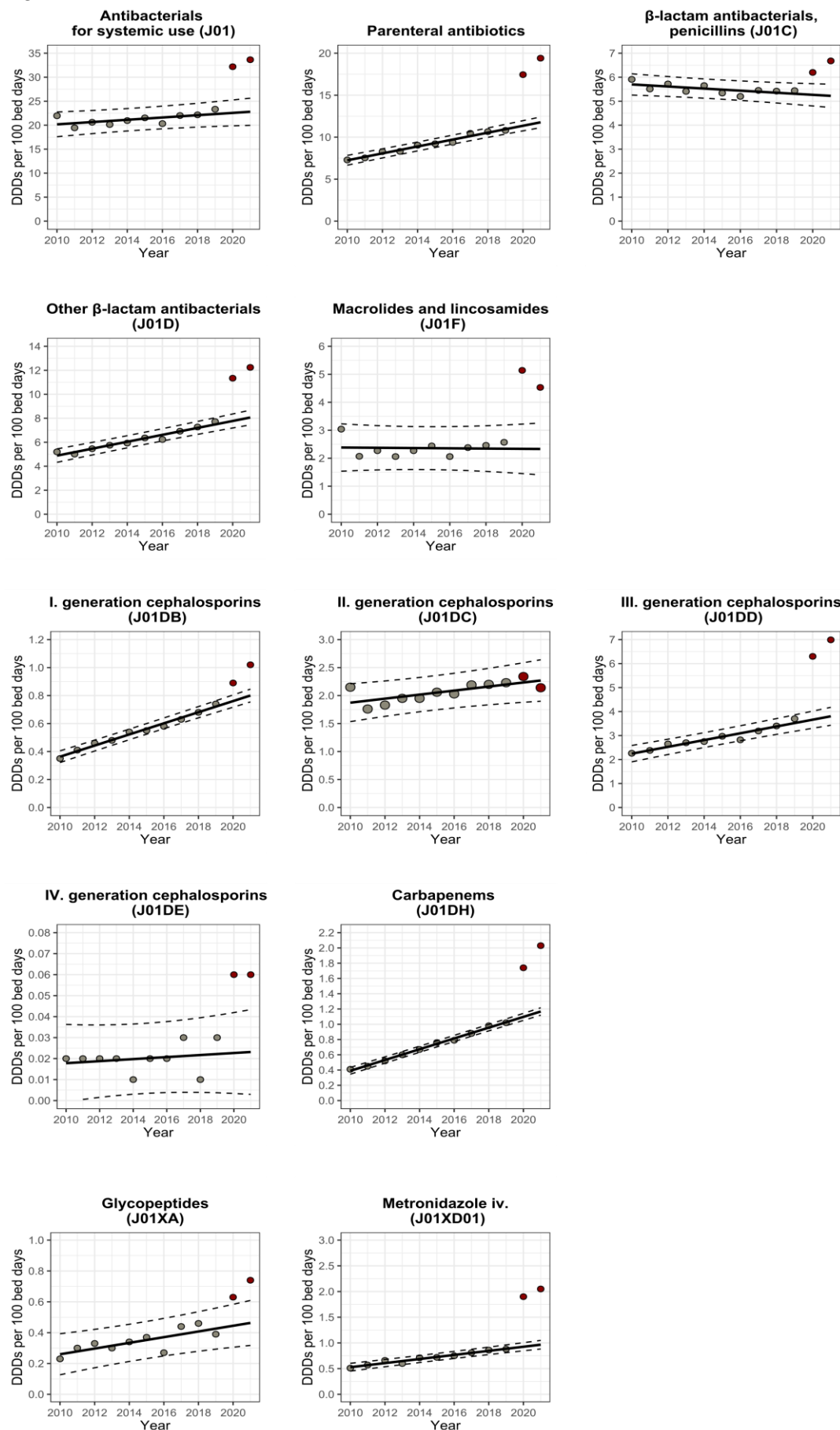

Supplement: Supplementary file 1 [file antibiotics-13-00102-s001.zip › antibiotics-2771503-supplementary.pdf]
